# Supplementary material for: Functional Importance of the Hydrophobic Residue 362 in Influenza A PB1 Subunit
Source: Viruses. 2023 Jan 30;15(2):396. doi: 10.3390/v15020396 (PMC9967172; doi:10.3390/v15020396)
Supplement: Supplementary file 1 [file viruses-15-00396-s001.zip › viruses-2181130-supplementary -revised.pdf]

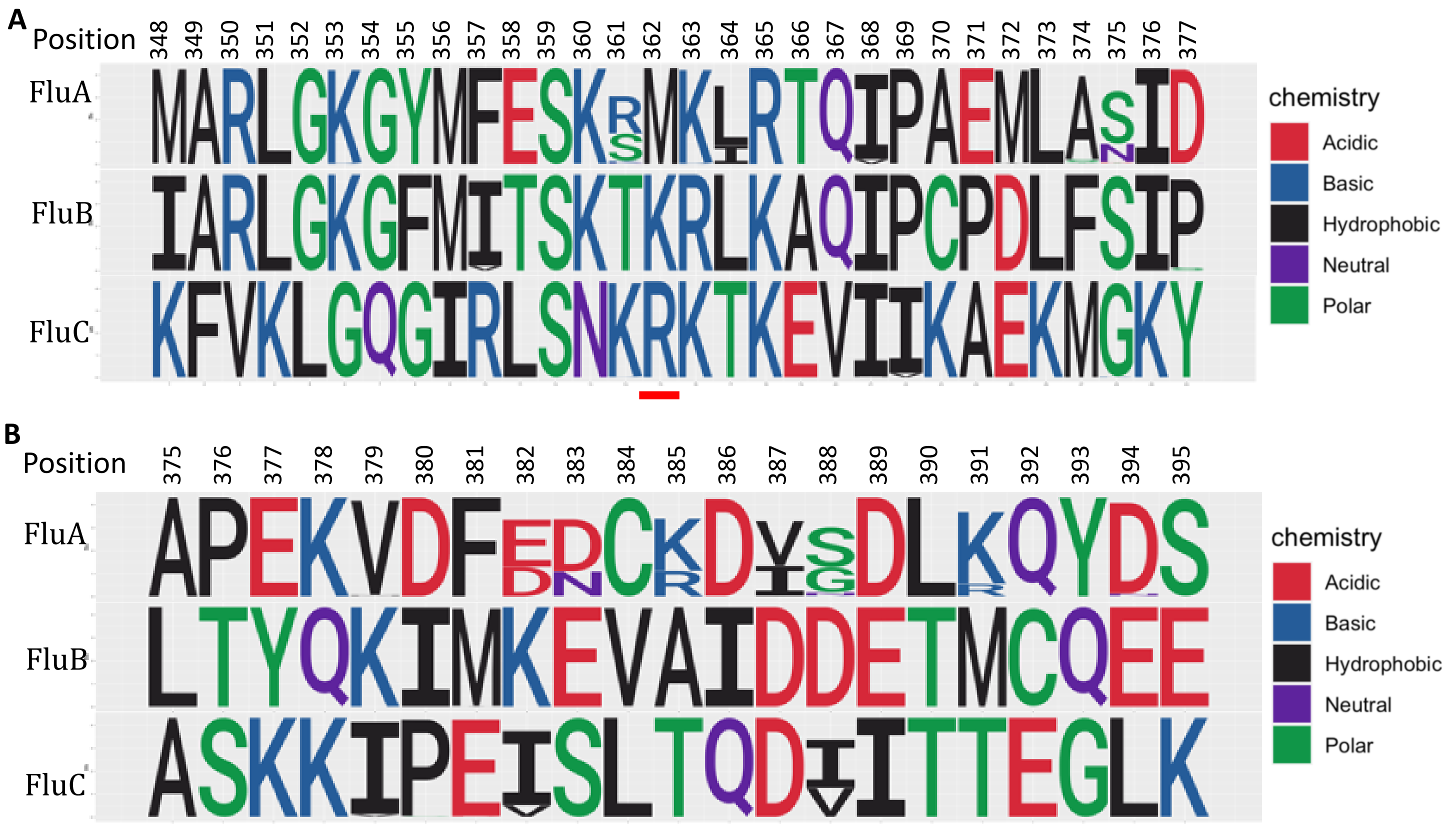

Supplementary Figure S1. Consensus sequence alignment along Influenza A, B and C virus at the PA-arch and PB1 β hairpin motifs

Sequence logo of (**A**) the β hairpin of PB1 (348-377) and (**B**) the PA-arch region wrapping the corresponding PB1 β hairpin (375-395). Sequences were aligned with all available sequences on the Influenza Data Base. Both subunits were aligned along IAV, IBV and ICV strains according to structural superposition. Residue numbers of the alignment was referenced to IAV. The residue underlined in PB1 panel indicates residue 362, which is the tip of the β hairpin.

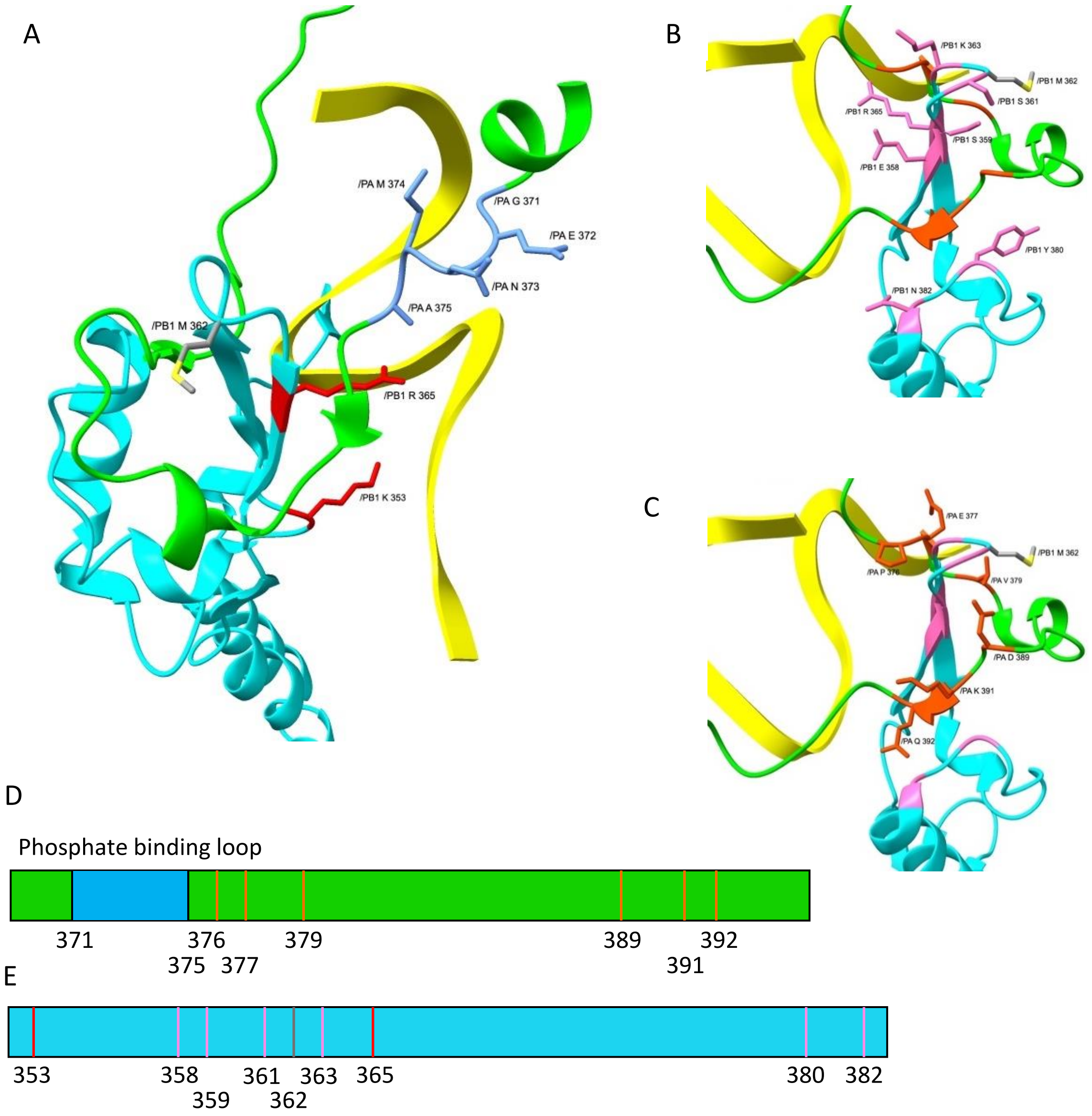

Supplementary Figure S2. Structural investigation of the PB1 β hairpin, PA-arch and the promoter (RCSB entry: 6RR7). (**A**) Promoter binding residues on both motifs were identified and colored. The promoter is colored in yellow. Promoter-interacting residues on PB1 were colored red and those on PA were colored cornflower blue. (**B**) and (**C**) indicates the residues involved in PB1-PA binding, in which those on PB1 were colored pink and those on PA were colored orange. Both figures showed the same snapshot of the polymerase, in which (**B**) showed sticks for PB1 residues and (**C**) showed sticks for PA residues. The schematic diagram (**D**), (**E**) represented the mentioned residues with the amino acid identified with corresponding color. PB1 362M were colored in grey in all three structures.

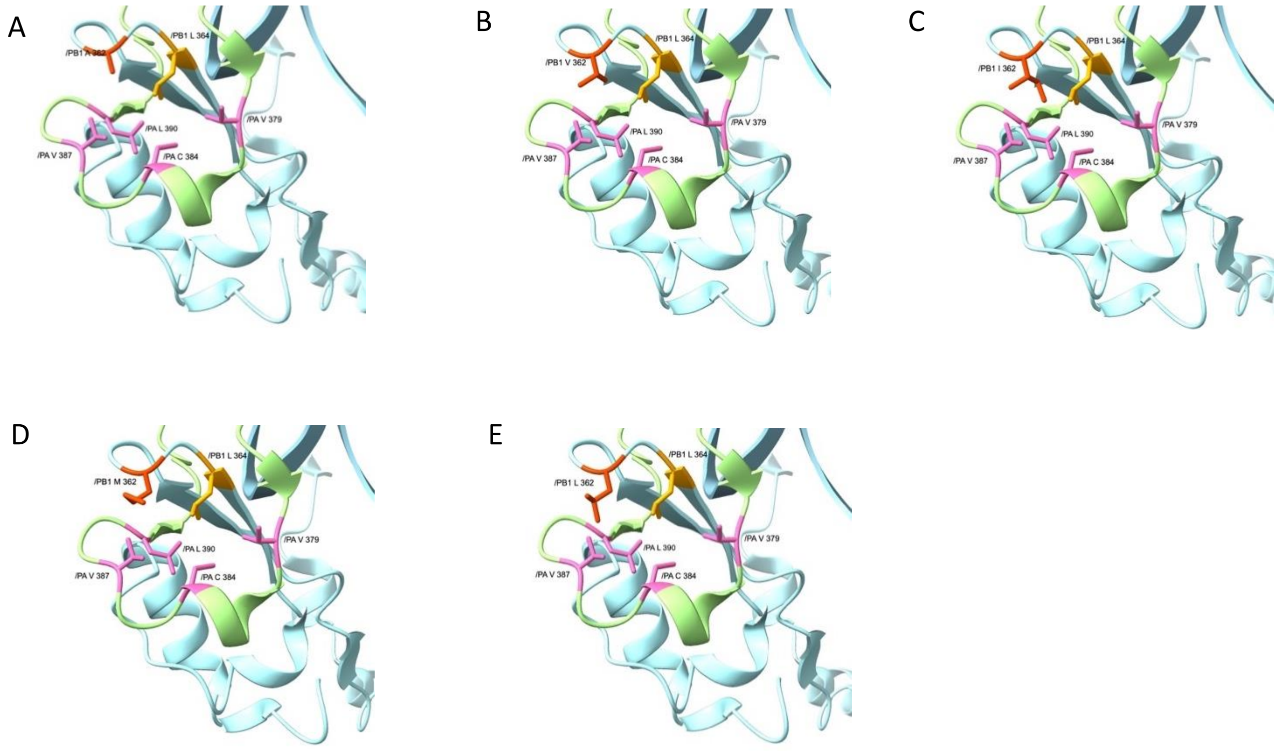

Supplementary Figure S3. Structural prediction of hydrophobic mutant PB1 362 (RCSB entry: 6RR7) Hydrophobic mutants of PB1 362 were predicted on the available structures. Variants of PB1 362 (**A**) Ala; (**B**) Val; (**C**) Ile; (**D**) Met and (**E**) Leu were predicted and colored in orange red, while PB1 364 was colored orange. Hydrophobic residues on PA-Arch motif were colored pink. As observed in (**A**) and (**B**), side chains of Ala and Val are too short to involve in the hydrophobic network, in which both mutants showed reduced polymerase activity in both WSN and 156/97 strains (Figure 1). In contrast, side chains of Ile, Met and Leu are long enough to integrate in the hydrophobic core, which all of the mutants showed the ability to replicate and transcribe in the RNP context (Figure 2). It is reasonable to predict that the size of the side chain of PB1 362 is correlated with the activity of the polymerase, where a longer chain results in higher activity.

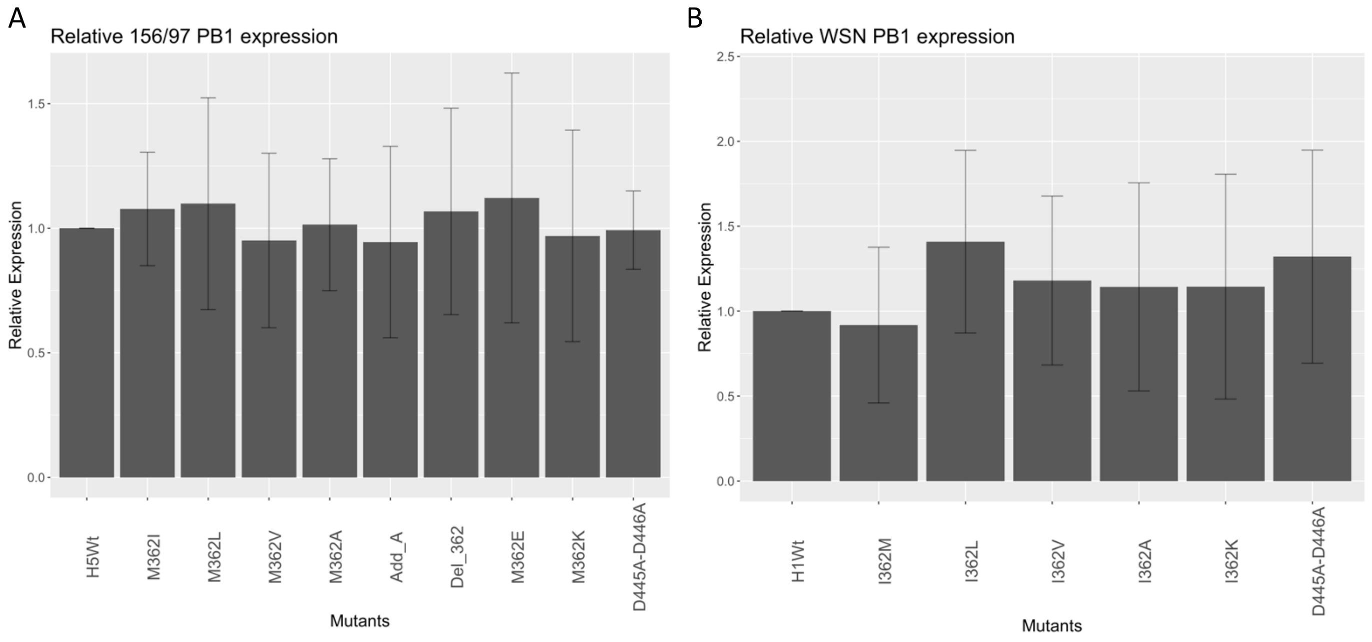

Supplementary Figure S4. Relative expression level of different PB1 mutants

To ensure the variation in polymerase activity was not affected by different PB1 expression, western blot analysis was performed after each independent experiment. PB1 expression was normalized to the corresponding β actin expression, analyzed by imageJ software (Schneider, Rasband and Eliceiri, 2012). Relative expression of 156/97 PB1 (**A**) and WSN PB1 (**B**) were plotted with reference to the wild type PB1. It is observed that PB1 expression showed slight variation among assays, but the degree of variation is far less than the differences in polymerase activity described.

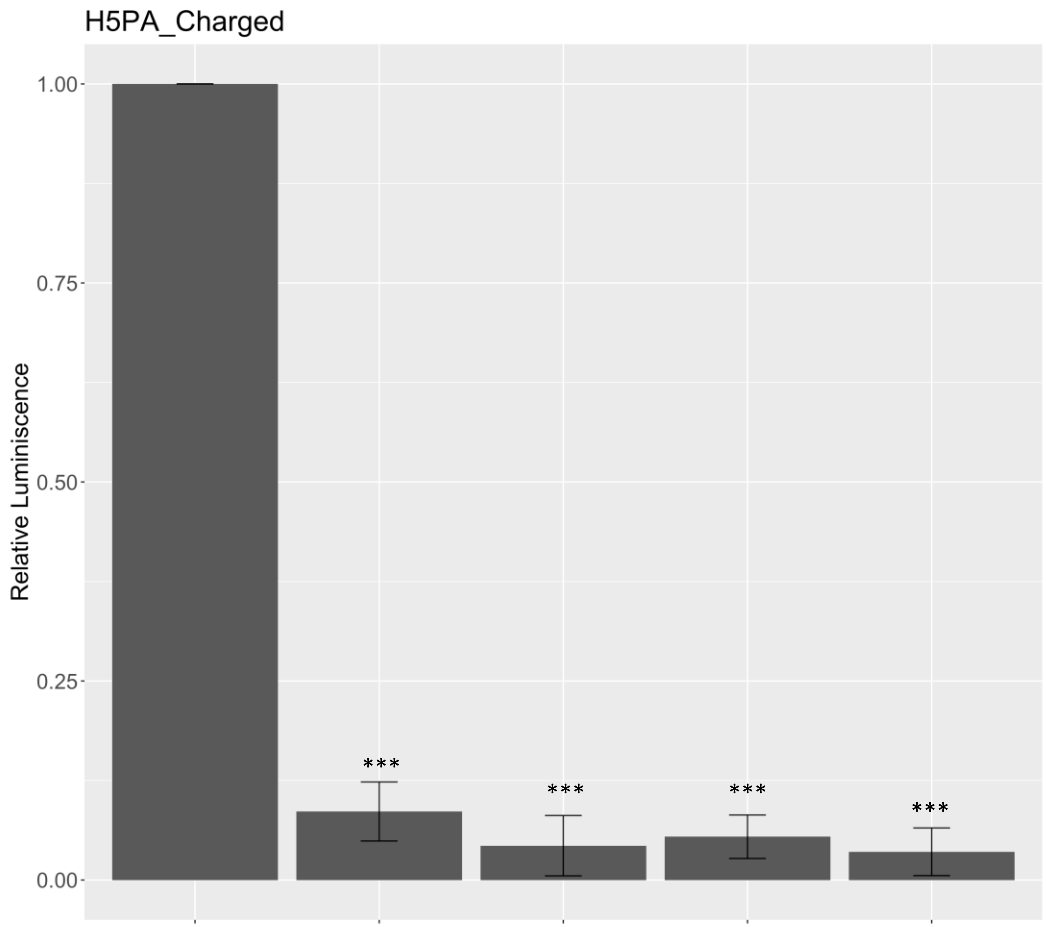

PB1	H5Wt				D445A-D446A
PA	H5Wt	V379D	C384D	V387D	H5Wt

Supplementary Figure S5. Minireplicon assay for H5PA charged mutants

On top of alanine screening, three amino acids on the PA-Arch of 156/97 PA were mutated into Asp, providing a charge to the hypothesized hydrophobic core. All charged mutants significantly reduced the polymerase activity reported by the minireplicon assay. Results of the mutants were normalized with the wild-type, and $PB1_{D445A\text{-}D446A}$ acted as the negative control. *P* values were determined by Student's T-test *** = *p* value < 0.001)

Host	Year	Position Variants (% out of total entry)						Total entry
		362M	362L	362I	362V	362A	362K	
Avian	Before 1980	553 (100%)	**	**	**	**	**	553
	1980-2000	1705 (99.243%)	3 (0.174%)	**	**	**	**	1718
	2000-2010	8418 (99.433%)	11 (0.130%)	1 (0.012%)	**	**	**	8466
	2010-2020	9612 (99.339%)	5 (0.052%)	2 (0.021%)	**	**	**	9676
Swine	Before 1980	152 (100%)	**	**	**	**	**	152
	1980-2000	207 (100%)	**	**	**	**	**	207
	2000-2010	1402 (97.8%)	10 (0.698%)	**	1 (0.070%)	**	**	1433
	2010-2020	4850 (99.733%)	13 (0.267%)	**	**	**	**	4863
Human	Before 1980	409 (99.272%)	**	1 (0.243%)	**	**	**	412
	1980-2000	1181 (100%)	**	**	**	**	**	1181
	2000-2010	8646 (99.827%)	1 (0.012%)	**	**	**	1 (0.012%)	8661
	2010-2020	21275 (99.991%)	**	2 (0.009%)	**	**	**	21277

Supplementary Table ST1. Residue 362 in PB1 sequences. Avian, Swine and Human virus isolates were taken into account according to the year of isolation. The number of total entries of the row is listed at the end and those with no entry were identified by **.

Primer Nature	Name	Sequence (5' - 3')
Reverse Transcription	Luc-mRNA	CCAGATCGTTCGAGTCGTTTTTTTTTTTTTTTTTATTATTAC
	Luc-cRNA	GCTAGCTTCAGCTAGGCATCAGTAGAAACAAGGGTGTTTTTTATTA
	Luc-vRNA	GGCCGTCATGGTGGCGAATGCTGAATACAAATCACAGAATCGT
	5s rRNA	TCCCAGGCGGTCTCCCATCC
qPCR	m/cRNA_q	CGTCGCCAGTCAAGTAACAACCGCG
	vRNA_q	CGAAATGTTCATACTGTTGAGCAA
	mtag	CCAGATCGTTCGAGTCGT
	ctag	GCTAGCTTCAGCTAGGCATC
	vtag	GGCCGTCATGGTGGCGAAT
	5s rRNA Fw	TACGGCCATACCACCCTGAA
	5s rRNA Rv	TAACCAGGCCCGACCCTGCT
Primer extension	Luc +ve	TCTTCATAGCCTTATGCAGT
	Luc -ve	CGATGACGGAAAAAGAGATC

Supplementary Table ST2. Primer list for qPCR and primer extension assays.
Primers for qPCR (including reverse transcription) were designed according to previous publication with slight modification (Kawakami *et al.*, 2011). The target gene was changed from segment 6 to luciferase, while the artificial tag remains the same. Both positive-sense target RNA shared the same forward primer in qPCR assays.
